# In This Issue

**Published:** 1994

## HOW WOMEN DRINK

Almost 60 percent of American women drink alcohol. Drs. Sharon C. Wilsnack, Richard W. Wilsnack, and Susanne Hiller-Sturmhöfel describe trends in women’s alcohol consumption over the past decades and compare the drinking behavior of women and men. For example, women abstain more and drink less than men. The authors also look at the drinking behavior of women of different ages and ethnic groups and describe risk factors that influence why and how women drink. (pp. 173–181)

## ALCOHOL, HORMONES, AND HEALTH IN POSTMENOPAUSAL WOMEN

Women today can expect to live one-third of their lives after menopause. Drs. Laura J. Tivis and Judith S. Gavaler review the effects of alcohol on hormone levels in this large population. The normal hormonal changes of menopause are associated with increased risk for certain diseases of the bones and heart. Both moderate and heavy alcohol consumption significantly affect hormone levels, potentially influencing the occurrence or progress of these diseases. Unknown factors—such as race, ethnic group, or diet—may modulate alcohol’s effects in different groups of women. Much research remains to be done to understand alcohol’s effects on hormone levels and women’s health. (pp. 185–188)

## GENETIC ASPECTS OF ALCOHOL USE AND ALCOHOLISM IN WOMEN

Patterns of alcohol use and alcoholism appear to be different between men and women. Drs. Dace Svikis, Martha L. Velez, and Roy W. Pickens explore the extent to which genetic factors may contribute to these gender differences. With respect to patterns of alcohol use, studies suggest that genetic factors play a role in determining quantity and frequency of alcohol consumption in both male and female twins. With respect to the clinical disorder of alcoholism, adoption and twin studies have supported a genetic contribution to male alcoholism, and several (but not all) studies have supported a genetic contribution to female alcoholism. Some of the most exciting work in alcohol research today is in this area. (pp. 192–196)

## ALCOHOL AND FEMALE SEXUALITY

Alcohol consumption can affect a woman’s sexual behavior and her sexual experiences. Dr. Jeanette Norris analyzes the link between drinking and a woman’s sexual activities. She describes the cycle that can develop as a result of drinking in women: Sexual dysfunction may be the cause of drinking, or conversely, excessive drinking may result in sexual dysfunction. Dr. Norris also discusses how alcohol consumption by women can contribute to sexual victimization or may result in high-risk sexual behaviors. (pp. 197–201)

## TREATMENT NEEDS OF WOMEN WITH ALCOHOL PROBLEMS

The risk of developing alcohol-related problems and the consequences of alcohol abuse and dependence differ between women and men. These differences may necessitate gender-specific (i.e., “women-sensitive”) treatment programs or specially targeted treatment components. Dr. Linda J. Beckman describes how treatment can be improved to serve women more effectively and indicates specific treatment components, such as counseling for sexual victimization, which should be included in women-oriented programs. (pp. 206–211)

## ALCOHOL AND OTHER DRUG ABUSE AMONG WOMEN

Of the men and women in treatment for alcohol and other drug abuse, approximately one-fourth of each group has abused multiple substances. And a higher proportion of women in treatment have abused illicit drugs. Dr. Barbara W. Lex describes the patterns and consequences of women’s alcohol and other drug use. She reviews hypotheses suggesting that gender differences are related to the variations in the patterns of substance abuse observed among women and men. (pp. 212–219)

## RISK FACTORS FOR DRINKING OVER A WOMAN’S LIFE SPAN

As a woman advances through her life, different element—such as her upbringing, relationships, or employment status—may increase her chance of developing alcohol problems. Dr. Edith S. Lisansky Gomberg reviews factors from various stages of women’s lives that have been associated with an increased risk of incurring alcohol problems. She suggests that for prevention efforts to be successful, they must focus on women in each age group who demonstrate such risk factors. (pp. 220–227)

## COGNITIVE DEFICITS IN ALCOHOLIC WOMEN

Long-term alcohol abuse can impair the brain’s intellectual and problem-solving functions. Dr. Sara Jo Nixon reviews the effects of alcohol consumption on brain function and structure in women. Alcoholic women experience significant deficits compared with nonalcoholic women in such areas as perceptual-motor skills, visual-spatial processes, learning/memory, and abstraction/ problem-solving. Alcoholic men and women are impaired to a similar degree, although women typically report shorter or less severe drinking histories. Data are contradictory with respect to alcohol-related changes in brain structure and electrical activity in alcoholic women. Research is needed to clarify the significance of these findings. (pp. 228–232)

## DRINKING AND ALCOHOL-RELATED PROBLEMS AMONG MINORITY WOMEN

Although relatively few epidemiologic studies have assessed drinking by minority women in the United States, patterns of use by these women have begun to emerge, many of which differ from those of white women. Dr. Raul Caetano compares drinking patterns among black, Hispanic, and white women in the United States. He also examines the factors (e.g., education and acculturation) that may influence alcohol use by these women. Although cultural differences may affect alcohol use by minority women, Dr. Caetano cautions that these factors probably are not as pivotal as once believed. (pp. 233–241)

## PREVALENCE OF DSM–IV ALCOHOL ABUSE AND DEPENDENCE: UNITED STATES, 1992

In NIAAA’s Epidemiologic Bulletin, Dr. Bridget F. Grant and colleagues use data from the recently concluded National Longitudinal Alcohol Epidemiologic Survey (NLAES) to report the prevalence of alcohol abuse and dependence in the United States. This study used criteria based on the most recent psychiatric classification of alcohol-related disorders from the *Diagnostic and Statistical Manual of Mental Disorders, Fourth Edition* (DSM–IV). More than 7 percent of adults met DSM–IV criteria for alcohol abuse, alcohol dependence, or both. Males were almost three times more likely than females to meet the criteria for alcohol abuse and/or dependence. However, the male-to-female ratio was lowest in the youngest age group among nonblack respondents, suggesting that the rates of these disorders in nonblack females may be catching up. (pp. 243–248)

